# Circulation of SARS-CoV–Related Coronaviruses and Alphacoronaviruses in Bats from Croatia

**DOI:** 10.3390/microorganisms11040959

**Published:** 2023-04-07

**Authors:** Dragan Brnić, Ivana Lojkić, Nina Krešić, Vida Zrnčić, Lea Ružanović, Tina Mikuletič, Martin Bosilj, Andrej Steyer, Tomislav Keros, Boris Habrun, Lorena Jemeršić

**Affiliations:** 1 Croatian Veterinary Institute, Savska cesta 143, 10000 Zagreb, Croatia; 2 Croatian Biospeleological Society, Demetrova 1, 10000 Zagreb, Croatia; 3 Institute of Microbiology and Immunology, Faculty of Medicine, University of Ljubljana, Zaloška cesta 4, 1000 Ljubljana, Slovenia; 4 National Laboratory of Health, Environment and Food, Grablovičeva 44, 1000 Ljubljana, Slovenia

**Keywords:** SARS-CoV, bat, sarbecovirus, RT-qPCR, NGS, sVNT

## Abstract

Bats are natural hosts of various coronaviruses (CoVs), including human CoVs, via an assumed direct zoonotic spillover or intermediate animal host. The present study aimed to investigate the circulation of CoVs in a bat colony in the Mediterranean region of Croatia. Guano and individual droppings from four bat species were sampled and tested with the E-gene sarbecovirus RT-qPCR, the pan-CoV semi-nested RT-PCR targeting the RdRp gene and NGS. Furthermore, bat blood samples were investigated for the presence of sarbecovirus-specific antibodies with the surrogate virus neutralization test (sVNT). The initial testing showed E-gene Sarebeco RT-qPCR reactivity in 26% of guano samples while the bat droppings tested negative. The application of RdRp semi-nested RT-PCR and NGS revealed the circulation of bat alpha- and betaCoVs. Phylogenetic analysis confirmed the clustering of betaCoV sequence with SARS-CoV–related bat sarbecoviruses and alpha-CoV sequences with representatives of the *Minunacovirus* subgenus. The results of sVNT show that 29% of bat sera originated from all four species that tested positive. Our results are the first evidence of the circulation of SARS-CoV–related coronaviruses in bats from Croatia.

## 1. Introduction

It is well known that bats are natural hosts of various coronaviruses (CoVs), including several SARS-CoV-1 and SARS-CoV-2–related viruses [1,2,3]; when it comes to SARS-CoV-2, no intermediate or reservoir species have been identified so far. The closest sequences to SARS-CoV-2, RaTG13, RmYN02 [4], and most recent BANAL-236 [5] were identified in horseshoe bats (genus *Rhinolophus*), which suggests that SARS-CoV-2 may have originated from an ancestral bat sarbecovirus. 

Horseshoe bats, populating Asia, Europe, and Africa, are considered to be natural reservoirs of sarbecoviruses that belong to the *Betacoronavirus* (betaCoV) genus. Most epidemiological studies of sarbecoviruses originate from China [2,4,6,7,8,9,10]; however, there is a growing number of reports from other countries [5,11,12,13,14,15,16,17], including Europe [18,19,20,21,22,23]. 

It has been proven that humans can transmit SARS-CoV-2 to other animals. Until 30 November 2022, World Organisation for Animal Health (WOAH) recorded 695 cases of SARS-CoV-2 infections in 26 animal species [24]. In the Netherlands and Denmark, SARS-CoV-2 was transmitted to farm-kept minks, then spread to other domestic mink populations, and then transmitted back to farm workers [23,25,26]. More recent cases include hamster-to-hamster-to-human transmission [27], deer-to-deer [28], and human-to-cat-to-human transmission [29]. Furthermore, it is known that some bat species are susceptible to SARS-CoV-2 infection, as was experimentally confirmed in fruit bats [30].

Situated between four diverse biogeographical regions and populated with thirty-three recorded bat species, Croatia is among the richest European countries in bat biodiversity [31]. All bat species in Croatia are insectivorous. They inhabit various environments, such as forests, underground sites, and also human settlements [32,33]. The most numerous Croatian bat species is the Schreiber’s bent-winged bat (*Miniopterus schreibersii*): its population is estimated at 95,000–150,000 individuals [31,34]. Members of this species are seasonal migrators (>350 km) [35,36], which is important for estimating the risk of transmission of viruses they carry, primarily diverse alphacoronaviruses (alphaCoVs), but also the novel lyssavirus (Lleida bat lyssavirus) and the novel ebolavirus (Lloviu virus (LLOV) [2]. The national population of the greater horseshoe bat (*Rhinolophus ferrumequinum*), a bat known to be a natural host of diverse alpha- and betaCoVs, is much smaller (13,000–20,000 individuals) [31,34]. These species are mainly sedentary, migrating between 10–60 km between summer and winter colonies [37]. Among other members of this genus, *R. blasii*, *R. euryale*, and *R. hipposideros* have also been recorded in Croatia. According to our previous serological findings, bats in Croatia are hosts of the European bat lyssavirus-1 (EBLV-1) [38]. Moreover, based on the virome investigation in Croatian bats, we previously discovered several sequences corresponding to alphaCoVs and a short sequence similar to the spike (S) gene of SARS-CoV [39]. 

Due to numerous findings of various sarbecoviruses in rhinolophid bats worldwide, decreased boundaries between human and animal habitats, and a high number of people being infected with SARS-CoV-2, our research was focused on discovering the possible circulation of sarbecoviruses related to the ongoing COVID-19 pandemics as well the diversity of other CoVs in a multispecies bat colony. 

## 2. Materials and Methods

### 2.1. Ethics Statement

All procedures, including capture and handling of bats and sample collection, were carried out in accordance with the ethical guidelines and the permit issued by the Croatian Ministry of Environmental Protection and Energy (Permit number: UP/I-612-07/20-48/140). The ministry guidelines on how to reduce the risk of SARS-CoV-2 transmission from humans to bats by cavers were strictly followed [40,41].

### 2.2. Bats Capturing and Sampling 

**Figure 1:**
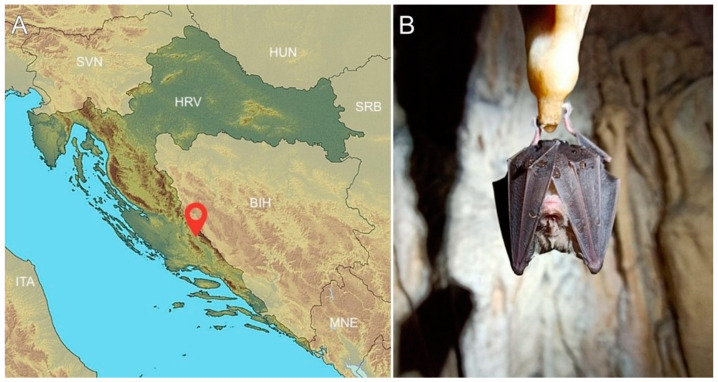
(**A**) Map of Croatia showing the location of the bat collection site. (**B**) Photograph of an *R. ferrumequinum* bat at the investigated location.

Bat capturing and sampling was performed by bat biologists at an underground site (cave) in Šibenik-Knin County (Mediterranean region) in October 2020 during two consecutive visits. The cave is located 2 km from the nearest human settlement (Figure 1) and inhabits 6000 individuals from 7 species (*M. schreibersii*; *Myotis blythii*; *Myotis capaccini*; *Myotis myotis*; *Rhinolophus blasii*; *R. ferrumequinum*; and *Rhinolophus hipposideros*). During our visit, four species of bats were captured: *M. schreibersii*, *M. capaccinii*, *M. myotis*, and *R. ferrumequinum*. Captures were facilitated by hand nets inside the colony dwellings during the day. During sampling, each bat was put into a separate cotton bag. Each bat was carefully taken out from the cotton bag, after which the sex (all adults) and species were morphologically determined by experienced chiropterologists (Table S1).

Blood samples (N = 38) from the uropatagial vein were collected from 13 *M. schreibersii*, 10 *M. capaccinii*, 8 *M. myotis*, and 7 *R. ferrumequinum* bats on filter papers (diameter = 0.6 cm) (Mini Trans-Blot; Bio-Rad, Hercules, CA, USA) using a 26-G needle (BD Microlance, Becton, Dickinson &Co. Ltd., Drogheda, Ireland) (Table S1). A maximum of 23 μL of blood was applied to each filter paper. Bat droppings (N = 11) (Table S1) were collected from bats defecating at the time of sampling and preserved in 500 μL of nucleic acid stabilization reagent (DNA/RNA Shield; Zymo Research, Irvine, CA, USA). Besides bat droppings, 19 guano samples were collected from 19 sites below the colonies using a DNA/RNA Shield collection tube with swab (Zymo Research, Irvine, CA, USA) or a dry cotton swab (Deltalab, Rubí, Barcelona, Spain). All bats were successfully released at the location of their capture after sample collection.

### 2.3. Nucleic acid Extraction and RT-PCRs

Bat droppings and guano samples preserved in DNA/RNA Shield were vortexed and centrifuged at 3000× *g* for 10 min. RNA was extracted from 140 μL supernatant samples using a QIAmp Viral RNA Mini Kit (Qiagen, Hilden, Germany), according to the manufacturer’s procedure. RNA extracts were stored at −80 °C until used. The samples were first tested using the E-gene Sarbeco real-time RT-PCR assay [42] (Table S2) and then using two real-time RT-PCR RdRp assays, one employing a pan Sarbeco probe and one employing a SARS-CoV-2–specific probe [42]. Additionally, the CDC N1/N2 real-time RT-PCR assay using an additional primer/probe set to detect the human RNase P gene [43] was used. The pan-CoV semi-nested RT-PCR targeting the RdRp gene followed by PCR product sequencing was also performed [44]. To monitor RNA extraction and ensure specimen quality, samples were also screened for the presence of the mammalian beta-actin gene [45]. All real-time RT-PCRs were performed on a Rotorgene Q (Qiagen, Hilden, Germany) or a QIAquant 96 5plex (Qiagen, Hilden, Germany) using the qScript XLT One-Step RT-qPCR ToughMix (Quanta Bio, Beverly, MA, USA) under conditions stipulated by the manufacturer. Conventional semi-nested RT-PCR was performed using the Superscript III One-Step RT-PCR system with a Platinum Taq DNA Polymerase (Thermo Fisher Scientific, Waltham, MA, USA) under conditions stipulated by the manufacturer. In each test, a positive human SARS-CoV-2 RNA (kindly provided by Dr. Ivan-Christian Kurolt, University Hospital for Infectious Diseases “Dr Fran Mihaljević”, Zagreb) was used as a positive control. Possible nucleic acid contamination was monitored using no template control (NTC). To test the specificity of the applied methodology, the collection of coronaviruses (Croatian Veterinary Institute, Department of Virology), including porcine epidemic diarrhea virus (PEDV), transmissible gastroenteritis virus (TGEV), bovine coronavirus (BoCV), and feline infectious peritonitis coronavirus (FeCV), were tested together with other samples using the E-gene real-time RT-PCR assay. PCR products were purified with ExoSAP-IT™ PCR Product Cleanup Reagent (Thermo Fisher Scientific, Waltham, MA, USA), and sequenced in both directions (Macrogen Europe, B.V., Amsterdam, Netherlands.).

### 2.4. Sequence and Phylogenetic Analysis

The partial RdRp reference genome sequences (Table S3) were downloaded from GenBank “https://www.ncbi.nlm.nih.gov/ (accessed on 7 February 2023)”. GISAID “https://www.gisaid.org/ (accessed on 18 June 2021), and NGDC “https://ngdc.cncb.ac.cn/gwh/Genome/ (accessed on 18 June 2021)”. The obtained RdRp sequences were aligned with the representative bat alphaCoV and betaCoV sequences, using MEGA11 (v. 11.013) [46]. Phylogenetic analysis was performed with neighbor-joining (NJ) and maximum likelihood (ML) method using the best-fit substitution model estimated by MEGA 11, the Tamura-Nei-with gamma-distributed rate heterogeneity (TN93 *+* G), and 1000 bootstrap replicates. The partial RdRp sequences of BetaCoVCro-BD10 (further in the text BD10), AlphaCoVCro-BD8 (BD8), and AlphaCoVCro-BS2 (BS2) were deposited in GenBank (accession Nos. MZ558550, MZ558551 and MZ558552, respectively).

### 2.5. Library Construction and NGS

Total nucleic acids were isolated from two guano samples BD10 and BD8 using a Maelstrom 9600 (Taiwan Advanced Nanotech Inc., Taoyuan City, Taiwan) automated nucleic acid extraction system loaded with OptiPure Viral Auto Plate reagents (Taiwan Advanced Nanotech Inc., Taoyuan City, Taiwan). After DNA removal with TurboDNase (Thermo Scientific, Waltham, MA, USA), cDNA was synthesized with the Maxima™ H Minus Double-Stranded cDNA Synthesis Kit (Thermo Scientific, Waltham, MA, USA) according to the manufacturer’s instructions using random hexamer primers for first-strand synthesis. After second-strand synthesis, dsDNA was purified using the GeneJET™ PCR Purification Kit (Thermo Scientific, Waltham, MA, USA) following the manufacturer’s protocol. The purified cDNA was stored at −20 °C until library preparation. After thawing, the purified cDNA was measured using the Qubit dsDNA HS Assay Kit (Invitrogen, Waltham, MA) and calibrated to 1 ng for further steps. The cDNA library was prepared with the Nextera XT DNA Library Preparation Kit (Illumina, San Diego, CA, USA) based on the Nextera XT DNA Library Prep Reference Guide (revised May 2019) using IDT™ for Illumina DNA UD Indexes (Ilumina, San Diego, CA, USA). For library cleaning, the protocol for 300–500 bp PCR amplicon input size was used, adding 90 µL of AMPure XP Beads to the amplified library. Library fragmentation quality and concentration were checked with the High Sensitivity DNA Kit (Agilent Technology, Santa Clara, CA, USA) on a 2100 Bioanalyzer (Agilent Technology, Santa Clara, CA, USA) and Qubit dsDNA HS Assay Kit (Invitrogen, Waltham, MA, USA), respectively. Prior to loading onto a NextSeq550 instrument (Illumina, San Diego, CA, USA), the library was normalized to 4 nM using the standard normalization method as described in the NexSeq System Denature and Dilute Libraries Guide (Illumina) (Revised December 2018). Loading of the normalized library into a NextSeq550 High Output Kit v2.5 (300 Cycles) cartridge was performed according to the NextSeq550 System Guide (Illumina) (Revised October 2021).

Raw reads were deposited in the NCBI SRA database (accession number: SRR21821664 in SRR21821663) under BioProject PRJNA887590.

### 2.6. Data Analysis

Compressed fastq files were first trimmed with Trimmomatic v0.39 [47]. Kraken2 2.1.2 and Bracken v2.6.2 were used for metagenomic analysis and Spades 3.15.4 for de novo assembly [48,49,50]. Quality assessment of the final assembly was performed with Quast 5.0.2 [51]. For automated implementation, all tools have been integrated into custom-made workflows using the Snakemake workflow management system [52]. 

### 2.7. Map to Reference Sequences

Reference genomes were chosen according to the results of the sequence analysis of obtained partial RdRp sequences. Sequence reads from sample BD10 were mapped to the SARS-CoV–related BM48-31/BGR/2008 genome sequence (GenBank accession No. GU190215), and from sample BD8 to BtMf-Alpha CoV/FJ2012 genome sequence (KJ473799) using Geneious Prime v.2022.2.1. (Biomatters Ltd., Auckland, New Zealand).

### 2.8. Serological Assays

Blood samples for serology were prepared as described previously [38,53]. First, filter papers were dried and incubated for 15 min at room temperature with 65 μL of the growth medium (DMEM with 10% FBS and 1% antibiotic/antimycotic solution) per piece, then centrifuged at the speed of 2000 rpm for 2 min. The prepared sera samples were transferred into new tubes and stored at −20 °C. To determine the presence of sarbecovirus-specific neutralizing antibodies against the S protein in prepared bat sera, the surrogate virus neutralization test (sVNT) using the cPASS SARS-CoV-2 Neutralization Antibody Detection Kit (Genscript Biotech, Leiden, The Netherlands), which measures antibody-mediated inhibition of SARS-CoV-2 RBD-ACE2 interaction, was used. The protocol was applied according to the manufacturer’s instructions. If present in the sample, neutralizing antibodies block the reaction between horseradish peroxidase (HRP)-labeled receptor-binding domain (RBD) and ACE2. The absorbance of the sample is inversely dependent on the titer of neutralizing antibodies in the tested samples. Samples showing an inhibition percentage equal to or higher than 20%, as the initial manufacturer-recommended positivity cutoff, are considered positive for SARS-CoV-2 neutralizing antibodies. Three SARS-CoV-2 antibody-positive and one SARS-CoV-2 antibody-negative human sera samples (kindly provided by Dr. Oktavija Đaković Rode, University Hospital for Infectious Diseases “Dr Fran Mihaljević”, Zagreb) were used as positive and negative controls, respectively. Additionally, SARS-CoV-2 antibody-negative animal sera samples previously proven negative [54] (four wild boar samples, two fox samples, and one jackal sample) served as negative samples.

## 3. Results

### 3.1. Sarbecovirus-Specific RNA Detected in Guano

The first-line screening assay targeting the E-gene revealed five positive samples with Ct values ranging from 27.70 to 33.59, all from guano (Table 1 and Table S4). Samples of bat droppings were negative. All negative controls were repeatedly negative, indicating the absence of contamination. Beta-actin was detected in all extracted RNAs, indicating that the host material was present. There were no cross-reactions with other tested coronaviruses from different animal species. The SARS-CoV-2 RdRp and N1/N2/human RNaseP real-time RT-PCR assays yielded negative results.

### 3.2. The Partial RdRp Gene Sequencing Revealed the Presence of Alpha- and BetaCoV Sequences 

Among five real-time RT-PCR positive guano samples, only three yielded positive results with pan-CoV semi-nested RT-PCR (Table 1 and Table S4). Obtained partial RdRp sequences revealed the presence of alphaCoVs in two samples, BD8 and BS2, 100% identical to each other and with a 98.06% identity to BtMf-Alpha CoV/FJ2012 (KJ473799), Neixiang-64 (KF294282), and BtMf-AlphaCoVHuB2013 (KJ473798) isolated from *Miniopterus fuliginosus* from China. Phylogenetically, using both methods, NJ and ML, BD8 and BS2 were clustered with other alphaCoVs from the *Minunacovirus* subgenus. Partial RdRp sequence from the sample BD10 was most similar (96.55%) to BatCoV/BM48-31/BGR/2008 from Bulgaria (GU190215) isolated from *R. blasii* and bat SARS-like CoVs Khosta-1 from Russia (MZ190137) isolated from *R. ferrumequinum*, followed by SarBatCoV1 (96.09%) from Italy (MG975784) from *R. ferrumequinum*. Compared with the SARS-CoV-2 sequence originating from a patient living in the same county where the bats were sampled (EPI_ISL_1591279|2021-02-27; Table S3), only 88.05% of identity was found.

**Figure 2:**
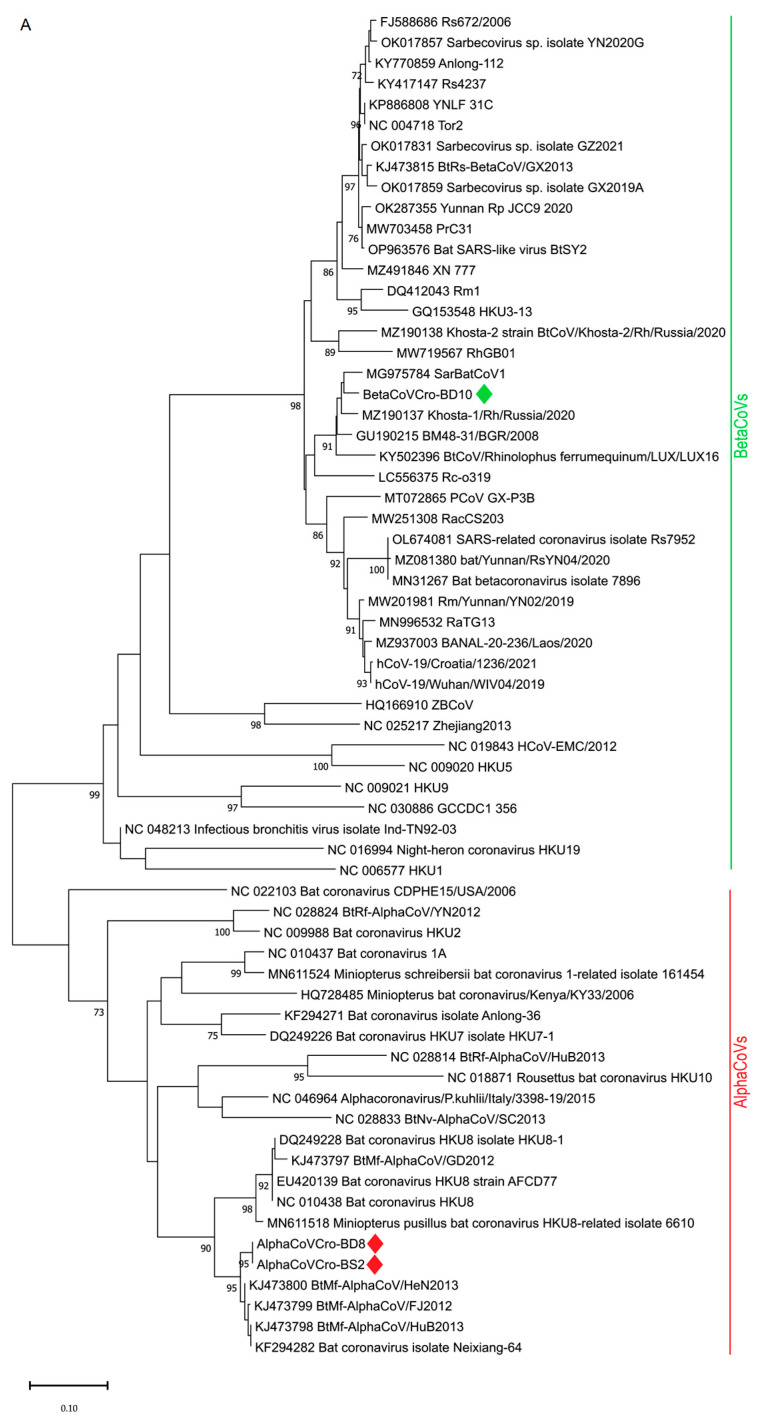

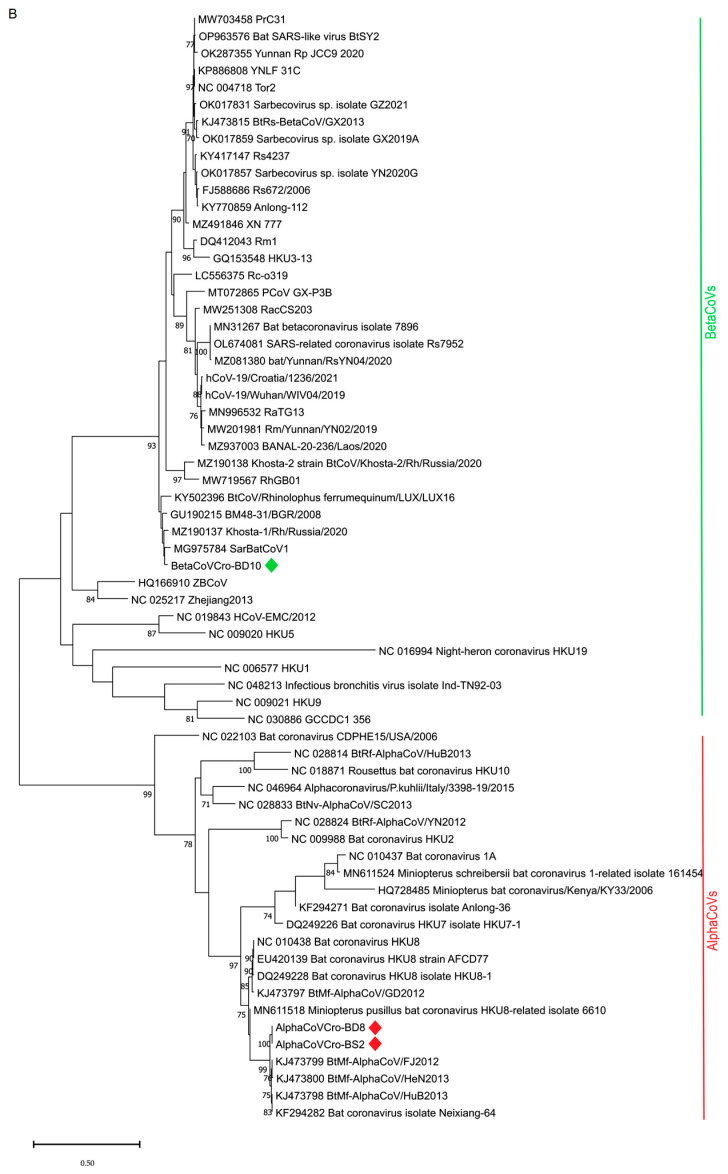
Phylogenetic analysis of 64 partial RdRp sequences of selected alpha- and betaCoVs (435 nt). The tree was inferred by neighbor-joining (**A**) and maximum likelihood method (**B**) using TN93 + G substitution model, the program MEGA11 and bootstrap values calculated from 1000 trees. The alphaCoV and betaCoV sequences obtained in this study are marked with red and green diamonds, respectively.

Both NJ and ML phylogenetic analysis ((Figure 2) of the partial RdRp sequences of betaCoVs revealed that BD10 clustered with bat SARS-CoV–related viruses from Europe (SarBatCoV1, Khosta-1, BatCoV/BM48-31/BGR/2008, BtCoV/LUX/LUX16_A_37/2016), sharing a common node with SARS-CoV-2 and SARS-CoV-2-related viruses.

### 3.3. Confirmation of Detected bat Alpha- and BetaCoVs with NGS

Reads were taxonomically classified with Kraken, and 14% of viral reads from the sample BD10 were assigned to sarbecoviruses. Among them, 1.068 reads were assigned to bat CoV BM48-31/BGR/2008. When mapped to the reference sequence, 1.481 reads from the sample BD10 were mapped to BM48-31/BGR/2008 genome sequence (GU190215). The resulting sequence had poor coverage (max = 24) and many gaps between covered parts. The longest part of the orf1ab sequence (nt 23427-24479, numbering from the BM48-31/BGR/2008 genome sequence) was 96% identical to BatCoV/BM48-31/BGR/2008 and BatCoV/BB9904/BGR/2008 (KR559017). The second longest stretch, 608 bp (nt 11390 to 11997) was 99% identical to BatCoV/BM48-31/BGR/2008.

Kraken identified 125 reads from the sample BD8 as *Miniopterus* bat coronavirus HKU8, 76 reads as SARS-CoV-1–related, and 10 reads as BM48-31/BGR/2008. When mapped to reference sequences, 451 reads from BD8 were mapped to BtMf-Alpha CoV/FJ2012 genome sequence (KJ473799) and 498 to BM48-31/BGR/2008 genome sequence.

Among de novo assembled contigs, several partial and full-length genomes, mainly from the order *Picornavirales*, were assembled, but will not be further analyzed within the present study.

### 3.4. Positive Serology in All Bat Species Using sVNT

Among 38 bat serum samples tested, 11 (28.94%) showed positive virus neutralization antibodies with low to moderate inhibition between 21.87 and 33.92 (Table S1), when tested using the sVNT assay. More males were positive (N = 8) than females. Among the positives, all species were represented. (Table 2 and Table S1).

## 4. Discussion

After the first two epidemics in this century, SARS and MERS, for which bat-borne transmission was confirmed, and especially during the current COVID-19 pandemic, the share of coronavirus research in bats has increased. Our study was conducted during the second wave of the COVID-19 pandemic. It included individual droppings, blood samples from four species of bats (*M. schreibersii*, *M. capaccinii*, *M. myotis*, and *R. ferrumequinum*), and guano samples. We used the opportunity to collect bat samples before their winter hibernation and, therefore, chose a Mediterranean location to be sure that bats were still active.

Here, we bring the first evidence of the circulation of SARS-CoV–related coronaviruses in bats from Croatia. This is also the first report on the presence of antibodies in bat species living on the European territory, detected by commercially available SARS-CoV-2–specific serological assay. No reverse spillover of SARS-CoV-2 from humans to bats was detected, even though the cave is relatively close to human settlements and open to visitors. That was confirmed by the negative results of applied SARS-CoV-2–specific real-time RT-PCR assays used in our study.

Positive results were obtained with the sarbecovirus-specific real-time RT-PCR assay (E-gene assay) and pan-coronavirus semi-nested RT-PCR. Furthermore, using partial RdRp sequencing and NGS, we discovered both bat sarbecovirus and bat alphaCoVs sequences.

Surprisingly, from the five samples that were positive with the E-gene Sarbeco assay, only three sequences were generated using the pan-coronavirus assay. Considering the confirmed limit of detection (LOD) for the E-gene assay (3.4 RNA copies per reaction), it can theoretically detect more positives than the pan-coronavirus assay (4–400 RNA copies per reaction) [42,44]. We have not examined the LOD for these assays in our laboratory conditions; we could only assume that our LOD for the E- gene assay is lower than for the pan-coronavirus assay. To further confirm the RT-PCR results, we prioritized NGS over virus isolation. Considering the quality of the samples, this proved to be the right decision.

The obtained sarbecovirus sequence BD10 clustered not only with other rhinolophid bat SARS-CoV–related viruses from Italy, Bulgaria, Russia, and Luxemburg (Figure 2A,B) but also with bat SARS-CoV–related Rc-o319 from Japan (Figure 2A). The greatest sequence similarity of our betaCoV BD10 with the bat SARS-CoV–related viruses from *R. ferrumequinum*, and the BatCoV/BM48-31/BGR/2008 isolated from *R. blasii* from Bulgaria is in accordance with the geographical range of these two species. *R. blasii* is distributed across the Mediterranean and overlaps with those of *R. ferrumequinum*, which range from southwest Europe to Japan [35]. Considering the overlapping habitats, a cross-species virus transfer is possible.

Unfortunately, we could not include in the analysis the sequences from Slovenia [20], Hungary [21], and most recent ones from Poland [22] because they were either too short or were located on a different part of the RdRp. The same applies to alphaCoVs. Our alpha CoV sequences clustered with those from *Miniopterus* bats from China. However, when we analyzed a significantly shorter fragment (296 nt), which corresponds to available sequences of European origin, 100% identity was found with alphaCoVs isolated from *M. schreibersii* from Spain (ON101717) and France (KY423482). We want to highlight this as the main drawback of analyzing bat CoVs in general because too few full-length sequences are deposited in publicly available sequence databases. A larger number of alphaCoV sequences from different parts of Europe would significantly contribute to the knowledge about the abundance of alphaCoVs carried by *Miniopterus* bats, which are long-distance seasonal migrants. Since that particular bat species was the most sampled species in our study, the detected alphaCoV probably originated from that species.

The finding of both bat alpha- and betaCoVs sequences in a colony was not surprising considering the number of different species co-roosting there. Co-infection of bats with multiple coronaviruses at the same time, or co-circulation of multiple virus genotypes within a roost, has been described previously [55]. Based on results obtained by Sarbeco E-gene real-time RT-PCR assay, we assumed that sarbecovirus RNA might be present in five guano samples. However, using the conventional semi-nested RdRp RT-PCR assay, alphaCoV RNA was identified in two (BS2 and BD8) out of five positive samples. Nevertheless, the presence of both sarbecovirus and alphaCoV RNA was confirmed by NGS in the BD8 sample. These findings further extend the knowledge of the circulation of diverse CoVs in bat colonies around Europe.

The interesting finding of the present study was the detection of SARS-CoV-2 neutralizing antibodies in eleven bats of all four sampled species. Bat blood collected on filter paper was previously successfully used for the detection of antibodies against the EBLVs [38,53]. In the present investigation, we used a commercially available sVNT. The applied test has already been used by Wacharapluesadee et al. [14] for the same purpose on *Rhinolophus* bats and pangolins in Southeast Asia, but with considerably fewer positives. The suitability of the applied serological assay on animal sera, except by the aforementioned [14], has been proven by Embregts et al. [56], and in our previous work [54]. It is also known that cPASS SARS-CoV-2 Neutralization Antibody Detection Kit cross-reacts with SARS-CoV-1 neutralizing antibodies, but not with other betaCoVs, alphaCoVs, or gammacoronaviruses [57,58]. Nevertheless, we are of the opinion that our findings and the findings of other groups applying the same sVNT should be interpreted with caution since confirmatory testing with the conventional plaque reduction neutralization test (PRNT) is considered warranted, especially if an equivocal area of binding inhibition % was introduced (15–35%) [59]. Since the results of binding inhibition % within the present study are mostly within an equivocal area, PRNT would most certainly prove beneficial. Unfortunately, in bat serology research, the sample volume is a limiting factor for the number of confirmatory assays that may be implemented. However, it is important to consider that within a multispecies colony, where bats roost in very dense clusters, they are exposed to various viruses [60]. The exposure can result in a detectable immune response, as we possibly detected here. Still, exposure to the virus does not necessarily mean that the virus has replicated in these individuals, especially in *Myotis* and *Miniopterus* bats.

There is no doubt that optimization and further validation of currently available serological protocols is necessary before large-scale bat testing is attempted. Nevertheless, we believe that screening for sarbecovirus RNA and antibodies in wildlife species represents a valuable approach for discovering yet unknown reservoirs or intermediate hosts. Finally, full-length or partial sequencing is still of the utmost importance in the diagnosis of emerging viruses in wildlife.

Since bats have been identified as natural reservoir hosts for several viruses related to emerging human viruses, it is of paramount importance to limit our contact with their habitats in order to prevent for a possibility of a new epidemic. It is equally important to prevent the transmission of SARS-CoV-2 from humans to bats or any other wildlife species in order to avoid the potential establishment of an animal reservoir.

## Figures and Tables

**Table 1:** The results of SARS-CoV-2 real-time RT-PCR and pan-CoV RT-PCR detection in bat droppings and guano samples.

	N	E-gene Real-Time RT-PCR	Pan-CoV RdRp RT-PCR
Bat droppings	11	0	0
Guano	19	5 (26.31%)	3 (15.78%)

**Table 2:** Results of the serological testing of bats for SARS-CoV-2 specific antibodies using cPASS SARS-CoV-2 Neutralization Antibody Detection Kit.

Bat Species	Number of Tested Bats	sVNT Positive Results
*M. schreibersii*	13	4 (30.76%)
*M. capaccinii*	10	3 (30.00%)
*M. myotis*	8	3 (37.50%)
*R. ferrumequinum*	7	1 (14.28%)
TOTAL	38	11 (28.94%)

## Data Availability

Sequences generated in this study are available in NCBI Genbank under accession numbers MZ558550, MZ558551, and MZ558552. Raw reads were deposited in the NCBI SRA database (accession number: SRR21821664 in SRR21821663) under BioProject PRJNA887590.

## Supplementary Materials

The following supporting information can be downloaded at: https://www.mdpi.com/article/10.3390/microorganisms11040959/s1, Table S1. Results of the serological survey of bats for SARS-CoV-2 neutralizing antibodies; Table S2. List of primers and probes used in this study; Table S3. A detailed list of sequences used in the phylogenetic analysis; Table S4. Results of molecular testing of guano swabs.
