# Supplementary material for: Circulation of SARS-CoV–Related Coronaviruses and Alphacoronaviruses in Bats from Croatia
Source: Microorganisms. 2023 Apr 7;11(4):959. doi: 10.3390/microorganisms11040959 (PMC10143505; doi:10.3390/microorganisms11040959)
Supplement: Supplementary file 1 [file microorganisms-11-00959-s001.zip › microorganisms-2274103-supplementary.pdf]

Table S1. Results of the serological survey of bats for SARS-CoV-2 neutralizing antibodies

Sample mark	Sample no.	Bat species	Sex	cPASS SARS-CoV-2 Neutralization Antibody Detection Kit (Inhibition %)
B1	1	*Miniopterus schreibersii*	m	-6,12/neg
B4	2	*Myotis capaccinii*	m	-9,81/neg
B6	3	*Myotis capaccinii*	m	3,65/neg
B7	4	*Myotis capaccinii*	m	16,71/neg
B8	5	*Miniopterus schreibersii* *	m	13,24/neg
B9	6	*Myotis capaccinii*	m	19,05/neg
B10	7	*Myotis capaccinii* *	f	N/A**
B12	8	*Miniopterus schreibersii* *	m	19,61/neg
B13	9	*Myotis myotis* *	f	6,03/neg
B14	10	*Rhinolophus ferrumequinum*	m	-15,62/neg
B15	11	*Rhinolophus ferrumequinum*	m	-10,87/neg
B16	12	*Rhinolophus ferrumequinum*	f	-1,45/neg
B17	13	*Myotis capaccinii*	m	5,59/neg
B18	14	*Miniopterus schreibersii*	m	14,12/neg
B19	15	*Miniopterus schreibersii*	m	28,55/pos
B20	16	*Rhinolophus ferrumequinum* *	f	10,69/neg
B21	17	*Miniopterus schreibersii*	f	18,26/neg
B22	18	*Myotis myotis*	m	-6,82/neg
B23	19	*Myotis myotis*	m	1,54/neg
B24	20	*Miniopterus schreibersii*	m	7,44/neg
B25	21	*Miniopterus schreibersii*	m	19,67/neg
B27	22	*Rhinolophus ferrumequinum*	m	26,70/pos
B28	23	*Miniopterus schreibersii* *	m	29,78/pos
B29	24	*Myotis myotis*	m	19,23/neg
B30	25	*Miniopterus schreibersii* *	f	14,83/neg
B31	26	*Rhinolophus ferrumequinum*	f	-0,92/neg
B32	27	*Myotis myotis*	f	-7,17/neg
B33	28	*Myotis myotis*	f	26,88/pos
B34	29	*Myotis myotis*	m	28,20/pos
B35	30	*Myotis myotis*	m	30,49/pos
B36	31	*Rhinolophus ferrumequinum*	f	18,43/neg
B37	32	*Myotis capaccinii* *	m	N/A**
B38	33	*Miniopterus schreibersii* *	f	N/A**
B39	34	*Miniopterus schreibersii*	m	25,30/pos
B40	35	*Myotis capaccinii*	f	24,59/pos
B41	36	*Myotis capaccinii*	f	-2,60/neg
B42	37	*Miniopterus schreibersii*	m	0,92/neg
B43	38	*Myotis capaccinii* *	m	N/A**
B44	39	*Myotis capaccinii* *	f	18,70/neg
B45	40	*Miniopterus schreibersii*	m	30,40/pos
B46	41	*Myotis capaccinii*	m	33,92/pos

B48	42	*Myotis capaccinii*	f	21,87/pos

* Bat dropping collected.
** Blood sample not collected.

Table S2. List of primers and probes used in this study.

Gene target/assay type	Primer and probe sequences	Reference
E/real-time	E_Sarbeco_F1:ACAGGTACGTTAATAGTTAATAGCGT<br>E_Sarbeco_R2: ATATTGCAGCAGTACGCACACA<br>FAM-ACACTAGCCATCCTTACTGCGCTTCG-BHQ1	Corman et al., 2020.
RdRp/real-time	RdRP_SARSr-F: GTGARATGGTCATGTGTGGCGG<br>RdRP_SARSr-R: CARATGTTAAASACACTATTAGCATA<br>RdRP_SARSr-P1 (pan Sarbeco):<br>FAM-CCAGGTGGWACRTCATCMGGTGATGC-BBQ<br>RdRP_SARSr-P2 (SARS-CoV-2):<br>FAM-CAGGTGGAACCTCATCAGGAGATGC-BBQ	Corman et al., 2020.
N1/N2 + Human RNase P gene /real-time	N1f: GACCCCAAAATCAGCGAAAT<br>N1r: TCTGGTTACTGCCAGTTGAATCTG<br>FAM-ACCCCGCATTACGTTTGGTGGACC-BHQ1<br>N2f: TTACAAACATTGGCCGCAAA<br>N2r: GCGCGACATTCCGAAGAA<br>FAM-ACAATTTGCCCCCAGCGCTTCAG-BHQ1<br>RNaseP F: AGATTTGGACCTGCGAGCG<br>RNaseP R: GAGCGGCTGTCTCCACAAGT<br>RNaseP: FAM-TTCTGACCTGAAGGCTCTGCGCG-BHQ1	Lu et al., 2020.
RdRp pan-CoV assay/semi-nested conventional	pan-CoV_outF: 5'- CCAARTTYTAYGGHGGITGG-3'<br>pan-CoV_R: 5'- TGTTGIGARCARAAYTCATGIGG-3'<br>pan-CoV_inF: 5'- GGTTGGGAYTAYCCHAARTGTGA-3'	Xiu et al., 2020.
Mammalian beta actin/real-time	ACT-1005-F: CAGCACAATGAAGATCAAGATCATC<br>ACT-1135-R: CGGACTCATCGTACTCCTGCTT<br>ACT-1081-HEX: HEX-TCGCTGTCCACCTTCCAGCAGATGT-BHQ1	Toussiant et al., 2007.

Table S3. Detailed list of sequences used in phylogenetic analysis.

AlphaCoV strain name	Accession no.	Bat species
Bat coronavirus CDPHE15/USA/2006	NC_022103	*Myotis lucifugus*
BtRf-AlphaCoV/HuB2013	NC_028814	*Rhinolophus ferrumequinum*
Rousettus bat coronavirus HKU10	NC_018871	*Rousettus aegypticus*
Bat coronavirus 1A	NC_010437	*Miniopterus sp.*
Miniopterus bat coronavirus HKU8	NC_010438	*Miniopterus sp.*
BtMf-AlphaCoV/FJ2012	KJ473799	*Miniopterus fuliginosus*

BtMf-AlphaCoV/HeN2013	KJ473800	*Miniopterus fuliginosus*
BtMf-AlphaCoV/HuB2013	KJ473798	*Miniopterus fuliginosus*
Neixiang-64	KF294282	*Miniopterus schreibersii*
HKU8-related isolate 6610	MN611518	*Miniopterus pusillus*
Isolate 161454	MN611524	*Miniopterus schreibersii*
BtMf-AlphaCoV/GD2012	KJ473797	*Miniopterus fuliginosus*
Kenya/KY33/2006	HQ728485	*Miniopterus inflatus*
Anlong-36	KF294271	*Miniopterus schreibersii*
HKU7	DQ249226	*Miniopterus magnater*
Bat-CoV/P.kuhlii/Italy/3398-19/2015	NC_046964	*Pipistrellus kuhlii*
BtNv-AlphaCoV/SC2013	NC_028833	*Nyctalus velutinus*
BtRf-AlphaCoV/YN2012	NC_028824	*Rhinolophus ferrumequinum*
Rhinolophus bat coronavirus HKU2	NC_009988	*Rhinolophus sinicus*
BetaCoV strain name	Accession no.	Bat species
Human coronavirus HKU1	NC_006577	*Homo sapiens*
Bat Hp-betacoronavirus/Zhejiang2013	NC_025217	*Hipposideros pratti*
Zaria bat coronavirus strain ZBCoV	HQ166910	*Hipposideros commersoni*
MERS-related coronavirus	NC_019843	*Homo sapiens*
Pipistrellus bat coronavirus HKU5	NC_009020	*Pipistrellus abramus*
Rousettus bat coronavirus	NC_030886	*Rousettus leschenaulti*
Rousettus bat coronavirus HKU9	NC_009021	*Rousettus leschenaulti*
Bat coronavirus BM48-31/BGR/2008	NC_014470; GU190215	*Rhinolophus blasii*
SARS coronavirus Tor2	NC_004718	*Homo sapiens*
Bat_SARS-like_coronavirus_BtCoV/Khosta-1/Rh/Russia/2020	MZ190137	*Rhinolophus ferrumequinum*
Bat_SARS_coronavirus_Rm1	DQ412043	*Rhinolophus macrotis*
Bat_SARS_coronavirus_HKU3-13	GQ153548	*Rhinolophus sinicus*
Bat_coronavirus_isolate_Anlong-112	KY770859	*Rhinolophus sinicus*
Bat_SARS-like_coronavirus_isolate_Rs4237	KY417147	*Rhinolophus sinicus*
Bat_SARS-like_coronavirus_YNLF_31C	KP886808	*Rhinolophus Ferrumequinum*
Bat_SARS_CoV_Rs672/2006	FJ588686	*Rhinolophus sinicus*

SARS-related_coronavirus_Rc-o319	LC556375	*Rhinolophus cornutus*
Pangolin_coronavirus_isolate_PCoV_GX-P3B	MT072865	*Manis javanica*
Bat_coronavirus_RaTG13	MN996532	*Rhinolophus affinis*
Bat_coronavirus_RacCS203	MW251308	*Rhinolophus acuminatus*
Bat_coronavirus_strain_BetaCoV/Rm/Yunnan/YN02/2019	MW201981	*Rhinolophus malayanus*
SarBatCoV1	MG975784	*Rhinolophus ferrumequinum*
LUX/LUX16_A_37/2016	KY502396	*Rhinolophus ferrumequinum*
Bat_coronavirus_isolate_PrC31	MW703458	*Rhinolophus blythi*
Betacoronavirus sp. RsYN04 strain bat/Yunnan/RsYN04/2020	MZ081380	*Rhinolophus stheno*
Rhinolophus bat coronavirus HKU2 isolate 6427	MN312267	*Rhinolophus affinis*
BANAL-20-236/Laos/2020	MZ937003	*Rhinolophus marshalli*
Betacoronavirus sp. isolate XN_777	MZ491846	*Rhinolophus pusillus*
Sarbecovirus sp. isolate YN2020G	OK017857	*Rhinolophus sinicus*
Sarbecovirus sp. isolate GZ2021I	OK017831	*Rhinolophus sinicus*
BtRs-BetaCoV/GX2013	KJ473815	*Rhinolophus sinicus*
Sarbecovirus sp. isolate GX2019A	OK017859	*Rhinolophus siamensis*
RhGB01	MW719657	*Rhinolophus hipposideros*
Bat CoV Rst7952	OL674081	*Rhinolophus stheno*
hCoV-19/Wuhan/WIV04/2019	EPI_ISL_402124|2019-12-30	*Homo sapiens*
hCoV-19/Croatia/1236/2021	EPI_ISL_1591279|2021-02-27	*Homo sapiens*

Table S4. Results of molecular testing of guano swabs

**Sample no**	**Guano/swab type**	**E-Sarbeco Real time RT-PCR**	**Pan-CoV assay/sequencing**
BD1	Dry swab	neg	
BD2	Dry swab	neg	
BD3	Dry swab	neg	

BD4	Dry swab	neg	
BD5	Dry swab	neg	
BD6	Dry swab	neg	
BD7	Dry swab	neg	
BD8	Dry swab	Ct=33,3	Pos/alphaCoV
BD9	Dry swab	neg	
BD10	Dry swab	Ct=27,7	Pos/betaCoV
BS1	DNA/RNA Shield / Swab	neg	
BS2	DNA/RNA Shield / Swab	Ct=32,14	Pos/alphaCoV
BS3	DNA/RNA Shield / Swab	neg	
BS4	DNA/RNA Shield / Swab	neg	
BS5	DNA/RNA Shield / Swab	Ct=33,22	Neg
BS6	DNA/RNA Shield / Swab	neg	
BS7	DNA/RNA Shield / Swab	Ct=33,59	Neg
BS8	DNA/RNA Shield / Swab	neg	
BS9	DNA/RNA Shield / Swab	neg	
